# A Unique Case of Metformin-associated Severe Lactic Acidosis Without Preexisting Renal Disease: Perspectives on Prolonged Dialysis and Education for Prevention

**DOI:** 10.7759/cureus.7564

**Published:** 2020-04-06

**Authors:** Kashif Abad, Cassandra Kien, Kavitha Ganta

**Affiliations:** 1 Nephrology, University of New Mexico, Albuquerque, USA; 2 Biological Sciences, Arizona State University, Tempe, USA; 3 Nephrology, Raymond G. Murphy Veterans Affairs Medical Center, Albuquerque, USA

**Keywords:** metformin, metabolic acidosis, acute kidney injury, lactic acidosis, mala, diabetes, hypertension, hemodialysis, chronic kidney disease

## Abstract

Metabolic acidosis is a common disorder defined by an imbalance in the body’s acid-base balance. Identifying the cause of acidosis is critical for its management. We describe a case of acute renal failure with lactic acidosis in a 69-year-old man who was taking metformin for type 2 diabetes. The patient presented with decreased urine output after two weeks of intermittent nausea and vomiting. During this time, the patient had continued to take limited fluids and medication, including lisinopril and metformin. Physical exam on initial evaluation was remarkable only for hypertension and minimal abdominal tenderness. However, laboratory tests revealed a severe lactic acidosis and renal failure with hyperkalemia. The patient had normal renal function and a normal urine albumin level three weeks prior. Broad-spectrum antibiotics and sodium bicarbonate were administered, followed by hemodialysis. During hemodialysis, the patient became hemodynamically unstable, requiring vasopressors. Post-dialysis, the lactic acidosis worsened, prompting the initiation of additional prolonged dialysis during the first hospital day. After the second lengthy dialysis, the patient’s condition improved significantly and he was discharged on hospital day 12, with the diagnosis of metformin-associated lactic acidosis (MALA) in the setting of acute tubular necrosis from gastrointestinal fluid loss accompanied by the continued use of an angiotensin-converting enzyme inhibitor. After discharge, his renal function returned to normal.

Severe lactic acidosis from metformin is relatively rare. Metformin has a large volume of distribution and accumulates in erythrocytes and intestinal cells, resulting in less efficient removal with dialysis and rebound lactic acidosis. Prolonged dialysis may be necessary for MALA to improve outcomes. Identifying metformin levels may help in diagnosis and management. However, the means to Identify metformin levels are not widely available. Patients receiving metformin should be counseled to stop metformin and seek medical care in the setting of illnesses. This is particularly important given the frequency of metformin prescription and the common use of renin-angiotensin system blockade in patients with type 2 diabetes, which increases the risk of kidney dysfunction.

## Introduction

Metformin (dimethyl biguanide) is the most commonly prescribed oral antihyperglycemic agent. It is considered the first-line drug for type 2 diabetes [1,2]. In the early twentieth century, metformin and other biguanides, linked to traditional herbal medicine Galega officinalis (rich in guanide), were found to lower blood glucose. However, the use of biguanides, primarily phenformin, to decrease blood sugar was largely stopped in the 1970s due to a high risk of lactic acidosis [1]. Nevertheless, the biguanide metformin, related to but much safer than phenformin, was continued to be used in Europe as a medication for diabetes and was introduced in the United States during the 1990s, where it gained widespread use [1].

The proximal small intestine is the main absorption site of metformin [3]. Metformin is not protein-bound and has a bioavailability of 50-60% with rapid distribution after absorption. It is excreted unchanged in the urine and is freely filtered and secreted by proximal tubules. The elimination half-life of metformin with normal renal function is around five hours. There is a correlation between increasing elimination half-life and decreasing renal function [4-6].

Metformin-associated lactic acidosis (MALA) is defined as an arterial pH of 7.35 or less and a lactate concentration of above 5 mmol/L in the setting of acute or chronic metformin exposure. MALA is diagnosed in the setting of risk factors for lactic acidosis, such as dehydration, heart failure, and cirrhosis, with increased production and/or decreased clearance of lactate [2,7,8]. However, with MALA, the pathophysiology of lactic acidosis is not defined. This is in contrast to metformin-induced lactic acidosis (MILA), which is usually seen in the setting of an acute metformin overdose or metformin use with acute renal failure where no other major risk factor for lactic acidosis exists [8]. MALA is extremely rare (≤10 events per 100,000 patient-years of exposure) but usually life-threatening, with a 45% mortality rate [2,9,10]. MILA, which has a lower mortality rate compared to MALA, is even rarer [11]. The differences between MILA and MALA may be often subtle.

## Case presentation

A 69-year-old Hispanic male with a history of type 2 diabetes mellitus presented to our emergency department (ED) complaining of decreased urine output for two days along with intermittent nausea and vomiting for two weeks. His past medical history included hypertension and coronary artery disease. Three weeks prior to presenting to the ED, our patient had undergone routine lab studies, which had documented normal kidney function with a serum creatinine level (Cr) of 0.97 mg/dL and no microalbuminuria. He had been taking metformin 850 mg three times a day and lisinopril 10 mg per day for more than a year, which he continued to take during his presenting illness. He was not taking any non-steroidal anti-inflammatory agents or other over-the-counter (OTC) medications. On initial evaluation, the patient was alert and oriented and in no distress. He was afebrile with a blood pressure of 152/69 mmHg, heart rate of 84 beats per minute, respiratory rate of 14 breaths per minute, and oxygen saturation of 96% on room air. Except for mild abdominal tenderness, his exam was unremarkable. 

Initial laboratory studies in the ED showed severe kidney injury with blood urea nitrogen (BUN) of 68 mg/dL and Cr of 12.2 mg/dL. Additionally, he was found to have a blood glucose level of 188 mg/dL, sodium level of 137 mmol/L, chloride level of 97 mmol/L, CO2 level of 8 mmol/L, potassium level of 6.2 mmol/L, and WBC count of 10,900 per uL with 83% neutrophils. Arterial blood gas revealed pH of 7.13 and partial pressure of carbon dioxide (PaCO2) of 21 mmHg. The anion gap (AG) was 32 mmol/L with a delta ratio of 1.3; lactate was 18.1 mmol/L. He had a pure high AG metabolic acidosis with high lactate levels. Renal ultrasound demonstrated kidneys of normal size and normal echogenicity without obstruction. Abdominal/pelvic CT was unremarkable. Supportive measures, including IV antibiotics, fluids, sodium bicarbonate as a drip infusion, and oral sodium polystyrene sulfonate were initiated. Bladder catheterization revealed less than 10 ml of urine. Urine microscopy was significant for brown muddy casts consistent with acute tubular necrosis (ATN). Electrocardiography results were normal.

**Figure 1:**
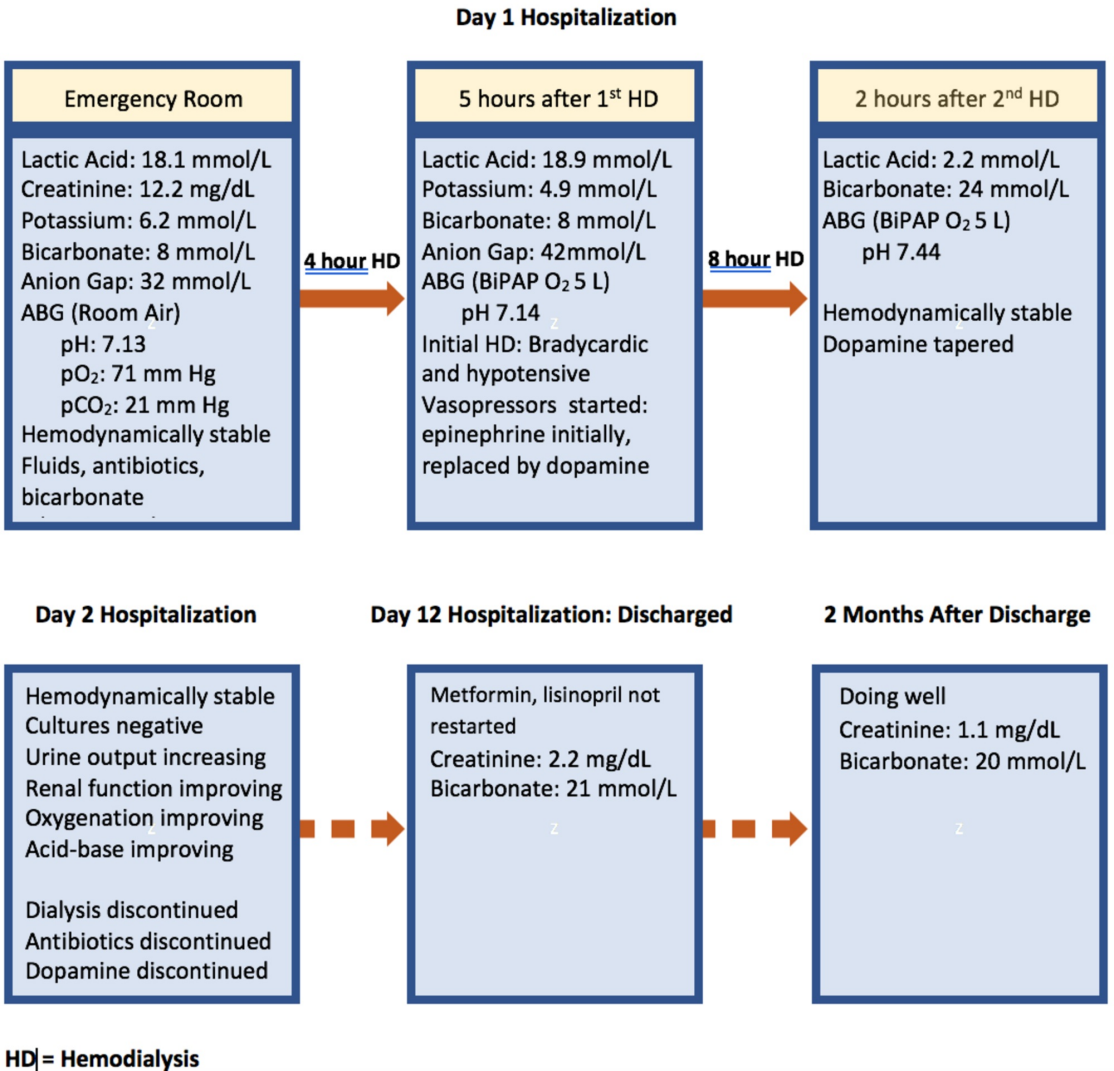
Hospital course and follow-up ABG: arterial blood gas

He was transferred to our intensive care unit, where urgent conventional hemodialysis with a blood flow of 250 ml/min was initiated for MALA and oliguric renal failure. Sodium bicarbonate drip infusion was discontinued before the initiation of dialysis. During the initial dialysis, he became hypoxic with a decreased level of consciousness and hemodynamically instability. His mean arterial pressure decreased to 50 mm Hg with a pulse rate of 43 beats per minute. He was treated with epinephrine, followed by dopamine. Bilevel positive airway pressure (BPAP) oxygen delivery was instituted. Dialysis was discontinued after four hours, and management was guided by his wishes regarding resuscitation as conveyed by his family. Initially, the directive was that there should be no further escalation of care. Repeat laboratory studies obtained five hours after dialysis revealed a continued metabolic acidosis with an arterial pH of 7.14, lactic acid of 18.9 mmol/L, and AG of 42. Dopamine was continued; blood pressure stabilized, and the patient became considerably more responsive. After a discussion with the patient and family, a second, slow, and low-efficiency dialysis was performed with a blood flow of 200 ml/min, lasting for eight hours. During the first hospital day, a total of 12 hours of dialysis was delivered. Following the second dialysis, lactic acid decreased to 2.2 mmol/L and arterial pH was found to be 7.44. On the second hospital day, the patient’s urine output increased; blood pressure was found to be 111/57 mmHg, and dopamine was discontinued. Oxygenation improved, and he was transferred out of intensive care on the fifth hospital day. He was discharged from the hospital on day 12 with a Cr of 2.1 mg/dl and CO2 of 21 mmol/L. Metformin and lisinopril had been discontinued. At the two-month follow-up, the patient had a Cr of 1.1 mg/dL. A summary of his course is shown in Figure 1.

## Discussion

Lactic acidosis is a rare but life-threatening consequence of metformin use. This case highlights the importance of recognizing that a patient with MALA or MILA may not appear to be critically ill on presentation. Rapid worsening of the patient’s condition can occur at any time. Metformin overdose may present with non-specific symptoms, including nausea, vomiting, diarrhea, and abdominal pain, as in our patient [3]. The most common complaints in MALA and MILA are gastrointestinal (GI)-related, followed by altered mental status, shortness of breath, and hypotension [3,12].

MALA and MILA are most likely to develop with renal insufficiency, liver disease, and in the setting of severe acute illnesses, which may result in decreased drug clearance [2]. Blood gas analysis and determination of the AG are key to identifying the nature of GI symptoms of uncertain cause in a patient taking metformin. A high AG metabolic acidosis in this setting is most likely due to lactic acidosis. In contrast, a non-AG hyperchloremic acidosis is most likely due to bicarbonate loss in diarrhea [13].

The pathogenesis of lactic acidosis from metformin is complicated and not completely understood. The mechanism is thought to be linked to impaired hepatic gluconeogenesis and compromised lactate clearance. A decrease in hepatic gluconeogenesis is thought to be due to the inhibition of the hepatic mitochondrial respiratory chain complex-1 by metformin. This inhibition results in the suppression of the production of adenosine triphosphate (ATP). ATP is an important and necessary high-energy source in the energy-demanding process of gluconeogenesis. A consequence of decreased hepatic gluconeogenesis is the accumulation of lactate, a precursor in this glucose-generating metabolic pathway. In addition, there is an accumulation of pyruvate, a substrate for the generation of lactate. Furthermore, metformin supports the conversion of glucose to lactate in the splanchnic bed of the small intestine, increasing the production of lactate. The end result is a toxin-induced type B lactic acidosis [2,5,14]. This is in contrast to the anaerobic type A lactic acidosis, the hallmark being tissue hypoxia. Most cases of MALA are characterized by contributions from both type A and type B lactic acidosis, whereas lactic acidosis in MILA would be considered to be due to metformin, a pure type B lactic acidosis [15].

Our patient showed no evidence of clinical shock or tissue hypoperfusion upon presentation, except for the findings consistent with ATN, suggesting metformin as the major contributing cause of lactic acidosis. The presence of an angiotensin-converting enzyme inhibitor interfering with autoregulation at the level of the renal arteriole in the setting of volume depletion is a plausible explanation for the development of ATN in our case. It is not clear if ATN predated the development of MALA or, less likely, that the GI symptoms represented MALA, followed by intravascular volume depletion and acute renal failure. Additionally, our patient made a rapid recovery after prolonged dialytic treatment without other significant interventions, supporting the idea that metformin was more likely the main cause of our patient's lactic acidosis. Although we cannot exclude dialysis as the cause of the patient's change in mental status, respiratory distress, and hemodynamic instability, this constellation of events is also consistent with metformin toxicity. Additionally, we cannot exclude the possibility of decreased cardiac contractility and hemodynamic instability from the development of a paradoxical intracellular acidosis as a result of bicarbonate administration as an IV drip followed by a bicarbonate dialysis bath. Because of the uncertainty of underlying factors that may have contributed to the severe lactic acidosis in our patient, we have elected to identify our case as MALA and not MILA.

Identifying metformin levels may help in diagnosis and management. A low or non-detectable metformin level, in our case, would have pointed to other causes of lactic acidosis. It may have also helped guide dialysis therapy. However, the means to Identify metformin levels are not widely available and were not readily available at our institution. Furthermore, some studies have shown that metformin levels vary significantly in MALA, partly as a result of confounding coexisting risk factors for lactic acidosis [2,16]. The inability to identify metformin levels is a limitation in our report and also played a role in this case being designated as MALA.

Like most poisonings, given the relative infrequency of use of dialysis as a mode of treatment, strong scientific evidence supporting the use of dialysis in the management of metformin toxicity with lactic acidosis is lacking [17]. In general, the risk vs. benefit, including the clinical condition of the patient, the poison's chemical properties and kinetics, and the availability of extracorporeal treatments including hemodialysis, peritoneal dialysis, and continuous renal replacement treatments, need to be considered. The Extracorporeal Treatment in Poisoning (EXTRIP) workgroup, by applying levels of evidence and expert opinion, have published guidelines to help inform decision-making in the use of invasive extracorporeal therapies for overdoses and poisonings, including the management of metformin toxicity [17]. In summary, there is no specific antidote for metformin toxicity. The mainstay of treatment, regardless of the cause, is supportive care. The conditions to initiate dialysis include lactic acid of >20 mmol/L, pH of ≤7.0, hypotension, decreased level of consciousness, kidney failure, and lack of response to supportive treatment. Dialysis should be continued until the pH is >7.35 and lactate is <3 mmol/L. Prolonged Intermittent hemodialysis is the preferred approach; however, continuous renal replacement treatment (RRT) has also been used successfully. It can be considered if conventional acute dialysis is not available or, depending on the medical center's capabilities, in the setting of hemodynamic instability [13,17]. The potential benefits of RRT go beyond metformin removal. They include rapid correction of acidosis, improvement in hyperlactatemia, correction of electrolytes, and other metabolic derangements in acute kidney injury [13]. Metformin sequestered in cells may rebound after dialysis. Careful monitoring is necessary after dialysis is discontinued, like in our patient, to ascertain if additional treatments are indicated [17].

Maintaining patient safety when prescribing metformin requires a better understanding and early recognition of the potential risk of MALA and MILA. Patients taking metformin may be susceptible to lactic acidosis during acute illness. This is especially true given the comorbid conditions among patients with diabetes and with the frequent use of renin-angiotensin system blockers in this population, increasing the risk of renal dysfunction. An educated and informed care team, including providers, pharmacists, nurses, diabetic educators, and ultimately, the patients themselves can help decrease the risk of MALA and MILA. A "sick day" plan to either adjust the dose or temporarily stop the use of metformin and other medications can be developed for the patient. Those taking metformin should be advised that vomiting and diarrhea could be early signs of lactic acidosis and prompt medical attention is needed when becoming acutely ill [18,19].

Our patient potentially would have benefitted from an action plan when he first became ill. He was on the maximum recommended dose of metformin as well as prescribed lisinopril. If evaluated early in his illness by an informed care team, decisions regarding medication modification may have altered his near-fatal course. In our case, we initiated a course of dialysis for four hours during which the patient became hemodynamically unstable with a decreasing level of consciousness. Although the family opted not to escalate care, the patient stabilized despite a worsening acidosis, and more aggressive management was instituted with prolonged, eight-hour dialysis. The early initiation of RRT with close monitoring for continued lactic acidosis and continuation of RRT is important in a subset of patients with MALA and MILA in order to decrease mortality. Additionally, it is important to manage other comorbid conditions, which may predispose patients to acute kidney injury and the development of MALA or MILA.

This case report was presented at the 2019 Western Medical Research Conference, Carmel, California.

## Conclusions

Early recognition of MALA is crucial for its management. Clinical findings at presentation, as in our patient, may correlate neither with the seriousness of the illness nor with the severity of abnormal laboratory findings. Patients with MALA or MILA who appear stable are at risk of rapid decline. Intensive care and nephrology consultation should be expeditious. Prolonged RRT may be required due to drug rebound. Our case underscores the importance of an informed care team and the education of patients taking metformin about the risk of MALA and MILA, as well as about the steps that can be taken to potentially prevent MALA and MILA. Urgent medical evaluation should be initiated if there is a possibility that MALA or MILA is developing in patients. Patients receiving metformin should be made aware of this.

## References

[1] Bailey CJ (2017). Metformin: historical overview. Diabetologia.

[2] DeFronzo R, Fleming GA, Chen K, Bicsak TA (2016). Metformin-associated lactic acidosis: current perspectives on causes and risk. Metabolism.

[3] McCreight LJ, Bailey CJ, Pearson ER (2016). Metformin and the gastrointestinal tract. Diabetologia.

[4] Graham GG, Punt J, Arora M (2011). Clinical pharmacokinetics of metformin. Clin Pharmacokinet.

[5] Rena G, Hardie DG, Pearson ER (2017). The mechanisms of action of metformin. Diabetologia.

[6] Lipska KJ, Bailey CJ, Inzucchi SE (2011). Use of metformin in the setting of mild-to-moderate renal insufficiency. Diabetes Care.

[7] Lalau JD, Race JM (2001). Lactic acidosis in metformin therapy: searching for a link with metformin in reports of 'metformin-associated lactic acidosis'. Diabetes Obes Metab.

[8] Vecchio S, Protti A (2011). Metformin-induced lactic acidosis: no one left behind. Crit Care.

[9] Kajbaf F, Lalau JD (2020). The prognostic value of blood pH and lactate and metformin concentrations in severe metformin-associated lactic acidosis. BMC Pharmacol Toxicol.

[10] Salpeter SR, Greyber E, Pasternak GA, Salpeter EE (2020). Risk of fatal and nonfatal lactic acidosis with metformin use in type 2 diabetes mellitus. Cochrane Database Syst Rev.

[11] Krowl L, Al-Khalisy H, Kaul P (2018). Metformin-induced lactic acidosis (MILA): review of current diagnostic paradigm. Am J Emerg Med.

[12] Vecchio S, Giampreti A, Petrolini VM (2014). Metformin accumulation: lactic acidosis and high plasmatic metformin levels in a retrospective case series of 66 patients on chronic therapy. Clin Toxicol (Phila).

[13] Nakamura A, Suzuki K, Imai H, Katayama N (2020). Metformin-associated lactic acidosis treated with continuous renal replacement therapy. BMJ Case Rep.

[14] Wang GS, Hoyte C (2018). Review of biguanide (metformin) toxicity. J Intensive Care Med.

[15] Lalau JD, Lacroix C, Compagnon P (1995). Role of metformin accumulation in metformin-associated lactic acidosis. Diabetes Care.

[16] Calello DP, Liu KD, Wiegand TJ (2015). extracorporeal treatment for metformin poisoning: systematic review and recommendations from the Extracorporeal Treatments in Poisoning Workgroup. Crit Care Med.

[17] Suzuki K, Okada H, Yoshida S (2020). Effect of high-flow high-volume-intermittent hemodiafiltration on metformin-associated lactic acidosis with circulatory failure: a case report. J Med Case Rep.

[18] Lea-Henry TN, Baird-Gunning J, Petzel E, Roberts DM (2017). Medication management on sick days. Aust Prescr.

[19] Weir MR, Fink JC (2014). Safety of medical therapy in patients with chronic kidney disease and end-stage renal disease. Curr Opin Nephrol Hypertens.

